# 

**Published:** 1895-03-16

**Authors:** 


**Notices of Births, Deaths, and Marriages** are inserted in this column at a charge of 2s. 6d. for an announcement not exceeding four lines.

**Notice to Advertisers and Subscribers.**

All Advertisements, Orders for Copies of The Hospital and Business Communications should be addressed to **The Manager, NOT TO THE EDITOR,** The Hospital, 428, Strand, London, W.C.

The Cost of Subscription, post free, is as follows:—Per week, 2½d.; per quarter, 2s. 8d.; per half-year, 5s. 3d.; per annum, 10s. 6d. Foreign:—Per Annum, 15s. 2d.; payable, in all cases, in advance.

NOTICE TO CORRESPONDENTS.

All MS., Letters, Books for Review, and other matters intended for the Editor should be addressed The Editor, The Lodge, Porchester Square, London, W.

The Editor cannot undertake to return rejected MS., even when accompanied by stamped directed envelope.

**"Burdett's Hospital Annual," 1895.**

Ready Early in March.

Sixth year of publication. About 1000 pp. Scarlet cloth, gilt lettered. Price 5s. A most complete guide to British, Colonial, Indian, and American Hospitals, Dispensaries, Nursing and Convalescent Institutions, and Asylums. A few copies of the "Annual" for 1894 may still be obtained on application to the Publishers, The Scientific Press (Limited), 428, Strand, London, W.C.

**NOTICE.—The Index for Vol. XVI. (ending September 24th, 1894) is on sale at the Office of this Journal, Price 2½d., post free.**

### SCRAPS AND GLEANINGS.

The Queen has sent a present of old linen to St. Thomas's Hospital.

The late Mr. John Halls, of Bury, has bequeathed £100 to the Huntingdon County Hospital.

Mrs. H. Maitland Everard, of Leamington, has just given £500 to the Spalding Johnson Hospital.

The Queen has sent her annual subscription of £21 to the East London Hospital for Children, Shadwell, E.

Her Majesty has been graciously pleased to accept a copy of Mr. F. J. Cross's work "Beneath the Banner," recently published by Messrs. Cassell and Co.

A donation of £20 has been made by the Grocers' Company to the Invalid Children's Aid Association, 18, Buckingham Street, Strand. Funds are needed to meet the many claims of the society.

The Worshipful Company of Leathersellers has, in addition to the 50 guineas already contributed to the General Fund of St. Thomas' Hospital, sent a donation, 200 guineas, to the special fund for opening the wards already closed.

The War Department have decided upon the erection of a station hospital, and accessory buildings at Shorncliffe Camp. The work will be carried out by private contract, a tender having been already accepted. The cost of the whole, it is understood, amounts to about £30,000.

The Ealing Cottage Hospital held its annual meeting recently. The statement of accounts proved to be fairly satisfactory. A slight decrease in the total income was recorded, but an increase in the numbers of subscribers has been entered. The institution is doing a good work in the district.

The sixty-ninth annual meeting of the Bath Royal United Hospital took place last week. The report stated that during the past year there had been no decrease in the demands on the hospital, and 1894 had been another witness to the need for such an institution; 1,383 patients had been treated during the year. The amateur theatrical week had benefited the hospital to the extent of £235, but there still existed a deficit on the balance sheet.

With the ultimate purpose of more fully advancing the science of stomatology, an academy has been established at Philadelphia. Here all encouragement will be provided for investigation and study of all matters connected with the mouth in health and disease. It is anticipated that the Association of Dental Practitioners, who have advanced this scheme, will finally be enabled to establish a museum and library in connection with the academy.

The Distribution Committee of the Hospital Saturday Fund has forwarded to the Chelsea Hospital for Women their cheque for last year's award (£95 1s.) which was withheld until the committee had been satisfied that the requirements and reforms mentioned in the official report of the Committee of Inquiry had been carried out. The committee arrived at their decision to forward the cheque at their last meeting, after receiving the report of a deputation appointed to visit the hospital.

### Books Received.

Cassell and Co.

"Year Book of Treatment."

Kelly and Co.

"Medical Directory."

Swan Sonnenschein.
"Darwinism and Race Progress." By J. B. Haycraft.

Scientific Press.
"Public Treatment of Pauperism." Report of International Congress of Charities.
"The Insane," "The Feeble-minded," "Criminals." Report of International Congress of Charities.

Balliere, Tindall, and Cox
"Ovarian Neuralgia and its Treatment." A. Rabagliati.

Isbister and Co.
"On Children." Anthony W. Thorold, D.D.
"On Being Ill." New edition. Anthony W. Thorold, D.D.
"On the Loss of Friends." New edition. Anthony W. Thorold, D.D.

John Ball and Sons.
"Difficulties of Prognosis in Insanity." Henry Sutherland.

Blackie and Son.
"Text-Book of Anatomy." Henry Edward Clark.

Henry Frowde.
"Hospital Service Book." C. Parkhurst Baxter, M.A.

**Periodicals and Pamphlets.**—Edinburgh Medical Journal, The Corpuscle, Medical Record, Science News, The Planter, Industries and Iron, Revue Générale des Sciences, The Therapist, Charity Organisation Review, Journal of the Anthropological Institute, British and Colonial Druggist, Excelsior, Magazine of James Murray's Royal Asylum, Perth, 1894, Chemist and Druggist, Penny Tales for the People, Sunday at Home, Leisure Hour, Girl's Own Paper, Boy's Own Paper, Clinical Sketches.

### The Student of Medicine.

### APPOINTMENTS.

A. Adams, M.B., C.M.Aberd., appointed Medical Officer to the Parochial Board of Alness, Ross shire. A. W. Aldrich, L.R.C.P., L.R.C.S.Edin., appointed Medical Officer to the Worlington District and Workhouse of the Mildenhall Union. H. T. Benson, L.R.C.P.Lond., L.S.A., reappointed Medical Officer for the Maxey District of the Peterborough Union. W. P. Briggs, L.R.C.P.Edin., L.F.P.S.Glasg., reappointed Medical Officer of Health to the Wigton Rural District. A. H. Carter, M.D.Lond., F.R.C.P., M.R.C.S.Eng., appointed Visiting Medical Officer to the Workhouse of the Parish of Birmingham. O. A. Dennis, L.D.S.R.C.S.I., appointed Dental Surgeon to the Nottingham General Dispensary. J. Ferguson, M.B., C.M.Glasg., appointed Visiting Physician to the Perth County and City Royal Infirmary. T. Geraty, L.R.C.P.I., M.R.C.S.Eng., reappointed Consulting Surgeon to the Nottingham General Dispensary. A.M. Gossage, M.B., B.Ch.Oxon., M.R.C.P.Lond., appointed Assistant Physician to Westminster Hospital. F. G. Haworth, M.B., C.M.Glasg., L.R.C.S., L.M.Edin., D.P.H.Camb., appointed Medical Officer of Health to the Darwen Urban Sanitary Authority; also Surgeon to the Darwen Division of the County Police. H. J. Heginbotham, L.R.C.P., L.R.C.S.Edin., L.F.P.S. Glasg., appointed Medical Officer to the Wymeswold District of the Loughborough Union. P. A. Kelly, L.R.C.P.Edin., L.M., L.R.C.S.I., appointed Surgeon to the Barry Island Railway and Harbour Works. D. S. Kennedy, M.B., C.M.Glasg., appointed Visiting Physician to the Perth County and City Royal Infirmary. Dr. S. K. Lister, appointed Medical Officer for the Terrington District of the Wisbech Union. C. A. Lumley, M.R.C.S.Eng., L.R.C.P.Lond., appointed District Surgeon to Idutywa, Transkei, Cape Colony. S. M. Magowan, M.B., B.Ch., B.A.O.R.U.I., appointed House Surgeon to the Belfast Royal Hospital. A. B. Mitchell, M.B.R.U.I., B.Ch., appointed Staff Assistant Surgeon

### THE STUDENT OF MEDICINE—*continued.*

to the Belfast Royal Hospital. W. Monckton, L.R.C.P.Edin., M.R.C.S.Eng., reappointed Medical Officer of Health to the Portishead Town Council. T. D. Pryce, M.R.C.S.Eng., L.S.A., reappointed Consulting Surgeon to the Nottingham General Dispensary. W. Robertson, M.D.Glasg., D.P.H., appointed Assistant Visiting Surgeon to the Perth County and City Royal Infirmary. C. F. Rudd, M.R.C.S.Eng., L.S.A., appointed Medical Officer for the Stalham District of the Smallburgh Union. R. Stirling, M.D.Edin., appointed Visiting Surgeon to the Perth County and City Royal Infirmary. J. W. Teale, M.A.Oxon, F.R.C.S.Eng., appointed to the Honorary Medical Staff of the Royal Northern Sea Bathing Infirmary, Scarborough. J. A. M. Thomson, L.R.C.P., L.R.C.S I., appointed Medical Officer of Health to the Bradford-on-Avon Rural and Urban Districts. Lilian Trewby, L.R.C.S., L.R.C.P.Edin., L S.A., late Medical Officer in Charge, Lady Dufferin Hospital, Shikarpur, Upper Sind, appointed Medical Officer in Charge, Lady Dufferin Hospital, Amraoti, Berar. E. B. Truman, M.D.St.And , M.R.C.S. Eng., reappointed Consulting Surgeon to the Nottingham General Dispensary. G. B. White, M.R.C.S.Eng., L.S.A., reappointed Consulting Surgeon to the Nottingham General Dispensary. Dr. H. W. L. Williamson, appointed Medical Officer for the Northern District of the Lancaster Union.

### VACANCIES.

#### Resident Medical Officers.

Belgrave Hospital for Children, Gloucester Street, S.W.—House Surgeon. Board, lodging, fuel, and light provided. Applications to the Honorary Secretary, at the hospital, before March 20th.

City of Glasgow District Lunacy Board.—Medical Superintendent of the new Asylum at Gartloch. Salary at the rate of £450 per annum, with free house, coal, gas, &c. Applications and testimonials to Mr. Dempster, Clerk to the Board, 318, Parliamentary Road, Glasgow, by March 25th.

General Hospital, Birmingham.—Two Assistant House Surgeons. Must possess surgical qualification and be registered. Appointment for six months. No salary, but residence, board, and washing provided. Applications and testimonials, with certificate of registration, to Howard J. Collins, House Governor, by March 30th.

Horton Infirmary, Banbury.—House Surgeon and Dispenser; duly qualified. Must be M.R.C.S Eng., and registered. Salary £60 per annum, with board and lodging. Applications and recent testimonials to Mr. C. H. Davids, Honorary Secretary, 21, Marlborough Road, Banbury, by March 23rd.

London Male Lock Hospital and Out-patients' Department, 91, Dean Street, Soho, W.—House Surgeon. Salary £50 per annum, with board, lodging, and washing. Must possess legally-qualified registered diplomas in medicine and surgery. Applications, with not more than three testimonials, to the Secretary by March 18th.

Metropolitan Hospital, Kingsland Road, N.E.—House Physician, House Surgeon, and Assistant House Surgeon. Appointments tenable for six months. The House Physician and House Surgeon will each receive a salary at the rate of £60 a year. Candidates must possess registered English medical and surgical qualifications. Applications and testimonials to Charles H Byers, Secretary, by March 18th.

Royal United Hospital, Bath.—House Surgeon, must be M.R.C.S.Eng. Salary £60 per annum, with board, lodging, and washing. Appointment for six months. Applications to W. Stockwell, Secretary-Superintendent, by March 20th.

Sussex County Hospital, Brighton.—Fourth Resident Medical Officer, doubly qualified, unmarried. Emoluments are board, residence, and washing, and a salary not exceeding £30 per annum. Applications to the Secretary by March 18th.

West London Hospital, Hammersmith Road, W.—House Physician and House Surgeon. Appointments tenable for six months, from April 1st. Board and lodging are provided. Candidates must be registered under the Medical Act. Applications and testimonials to R. J. Gilbert, Secretary-Superintendent, by March 20th.

#### Non-Resident Medical Officers.

Hospital for Sick Children, Great Ormond Street, Bloomsbury, W.C.—Medical Registrar and Pathologist. Appointment for one year. Honorarium 50 guineas. Applications to the Secretary by March 26th.

London Hospital, Whitechapel, E —Surgical Registrar, from April 1st. Salary £100 per annum. Applications and testimonials to G. Q. Roberts, House Governor, by March 29th.

Radcliffe Infirmary, Oxford.—Honorary Physician. Candidates must be legally qualified to practice as Physician in England. Testimonials to A. C. Virgo, Secretary, by March 25th.

#### Examining Board in England by the Royal Colleges of Physicians and Surgeons.

The following gentlemen having passed the necessary examinations, have been admitted, by the two Royal Colleges, Diplomas in Public Health: E. Ferrand (Surgeon-Major Madras Army), M.R.C.S.Eng., L.R.C.P.Edin.; J. W. Gill, L.R.C.P.Lond., M.R.C.S Eng.; R. T. Meadows, M.D. and C.M.Edin.; S. G. Moore, M.B. and Ch.B.Vict.; H. W. Newton, L.R.C.P.Lond., M.R.C.S.Eng.; A. Paine, M.B. and B.S. Lond.; E. H. Helby, L.R.C.P.Lond., M.R.C.S.Eng.; G. Jones, M.B. and B.Ch.Oxon., L.R.C.P.Lond., M.R.C.S.Eng.; R. F. H. Jones, L.R.C.P. Lond., M.R.C.S.Eng.; C. Paine, M.B.Lond.; W. Russell, M.B. and C.M.Aberd.; H. Simmons, M.B. and B.S.Durh., L.R.C.P.Lond., M.R.C.S.Eng.; G. A. Wilkes, L.R.C P.Lond., M.R.C.S.Eng.

### CHARTS

PRICES (Post or Carriage Paid)—

1,000, 25s.; 500, 13s. 6d.; 250, 7s. 6d.; 100, 3s. 6d.; 50, 2s.; 25, 1s. 3d.

TEMPERATURE, NURSING & DIET CHARTS,

As used in almost all our principal Hospitals.

THE BEST AND CHEAPEST PUBLISHED.

WODDERSPOON & CO.,

7, SERLE ST., LINCOLN'S INN, W.C.

### THE NURSES' BOOKSHELF.

Second Edition. Demy 8vo, 200 pp., profusely Illustrated with Copyright Portraits and Drawings. Price 2s. 6d. post free.

**How to become a Nurse: and How to Succeed.** A complete guide to the nursing profession for those who wish to become Nurses, and a useful book of Reference for Nurses, who have completed their training and seek employment. Compiled by HONNOR MORTEN, Author of "Sketches of Hospital Life," "The Nurses Dictionary," &c.

"To those who are frequently appealed to by girls or young women, as to the steps they should take to become nurses, this book of Miss Morten's must prove a perfect godsend."—*British Medical Journal.*

Second and Revised Edition. Demy 16mo, suitable for the apron pocket, handsomely bound in cloth, 140 pp. Price 2s.; in handsome leather, gilt, price 2s. 6d. net.

**The Nurses' Dictionary of Medical Terms and Nursing Treatment.** Compiled for the use of Nurses, by HONNOR MORTEN. Containing descriptions of the principal Medical and Nursing Terms and Abbreviations, Instruments, Drugs, Diseases, Accidents, Treatments, Physiological Names, Operations, Foods, Appliances, &c., &c., encountered in the Ward or Sick-room.

"Should be at the disposal of every nurse."—*Birmingham Medical Review.*

For Use in Hospital as well as Private Nursing.

Second Edition. Demy 16mo (for the apron pocket), 72 pp., strong boards, price 6d., or 4s. 6d. per doz., post free.

**"The Hospital" Nurses' Case Book.** A Book of Tables specially prepared for use by Nurses in the Ward and in the Sick Room. Twenty-Six complete Tables, each being ruled to last one week, for keeping an exact record of a Patient's condition. Nurses should combine together and order parcels of over a dozen to profit by the special discount terms—*i.e.*, 4s. 6d. per dozen, post free.

Second Edition. Enlarged, demy 8vo, profusely Illustrated, nearly 200 pp., cloth. Important Text-book for Asylum Attendants. Price 2s. 6d., post free.

**Mental Nursing.** A text-book specially designed for the instruction of Attendants on the Insane. By WILLIAM HARDING, M.B. The want of a complete book for the instruction of Asylum Attendants has been long felt, and it is with confidence that the publishers recommend this work to the managers of all Institutions for the Insane.

Important New Handbook on Ophthalmic Nursing.

200 pp., Crown 8vo., cloth, 3s. 6d. Profusely illustrated with Original Drawings, with list of Instruments required, and a Glossary of Terms.

**Ophthalmic Nursing.** By SYDNEY STEPHENSON, M.B., F.R.C.S.E., Surgeon to the Ophthalmic School, Hanwell, W., &c., &c. This is a valuable contribution to Nursing literature on a subject hitherto never handled with special reference to Nursing. On account of its compact and clear arrangement, and its numerous illustrations, it is specially recommended to the use of the Nursing Profession.

"May very advantageously be studied by nurses and clinical clerks, or dressers who are about to enter the Ophthalmic service of a hospital."—*The Lancet.*

Demy 8vo, nearly 200 pp., cloth gilt, profusely Illustrated with over 70 special cuts. Price 6s., post free.

**The Art of Massage.** By A. CREIGHTON HALE. A complete Text-book of this valuable art. The most practical, simple, and generally useful work published on the subject. With fully illustrated chapters on the Anatomy of the Human Body.

"The latest and most complete book we know on Massage."—*Queen.*

Just published, crown 8vo, imitation cloth, 5s. With 10 plates and many Illustrations in the Text, and Fac-simile Autograph Letters from the late Sir Andrew Clarke, M. Pasteur, &c.

**Infant Feeding by Artificial Means:** A Scientific and Practical Treatise on the Dietetics of Infancy. By S. H. SADLER, Author of "Suggestions to Mothers," "Management of Children," "Education."

Sixth Edition, profusely Illustrated with over 100 cuts; carefully revised and bound in cloth, crown 8vo, about 400 pp., price 3s. 6d. (post free).

**Theory and Practice of Nursing.** A Text-book for Nurses. By PERCY G. LEWIS, M.D., M.R.C.S., L.S.A. Always a standard work on Nursing; it now occupies the position of a classic, being used throughout the world in Training Schools and by Private Students.

"We warmly recommend it as a nursing manual . . . full of useful hints and information."—*Birmingham Medical Review.*

"Full of interest and instruction . . . cannot fail to assist nurses in their work."—*British Medical Journal.*

Demy 8vo, cloth gilt, 260 pp., price 3s. 6d.

**The Art of Feeding the Invalid.** By a Medical Practitioner and a Lady Professor of Cookery. A popular treatise on the most frequent disorders; with detailed lists of suitable diet and original recipes for daily use. This important work has been undertaken to supply a want long felt by Matrons and Lady Superintendents in Hospitals and Institutions of all kinds where invalids are treated, as well as in private households. It is of the greatest value to Nurses in private practice.

Third Edition, enlarged and partly re-written, cloth gilt, crown 8vo, about 200 pp., price 2s. 6d.

**Hospital Sisters and their Duties.** By EVA C. E. LÜCKES, Matron to the London Hospital. The favourable reception accorded to the first two editions of this useful and interesting work, and the regular demand for copies of it, encourage the publishers to place this third edition, greatly improved and enlarged, before the Institutions. The experience and position of its author entitle it to authority, and every Matron, Sister, or Nurse who aspires to become a Sister should read the advice and information contained in its pages.

Demy 8vo, 200 pp., cloth boards, copiously Illustrated, price 3s. 6d. (post free).

**Surgical Ward-work and Nursing.** By ALEXANDER MILES, M.D. (Edin.), C.M., F.R.C.S.E. A practical manual of clinical instruction for Nurses and Students in the Wards. Concisely, though comprehensively treated.

Crown 8vo, 500 pp., Second Edition, 7 Plates, 18 Illustrations, Charts, &c., price 7s. 6d. net.

**Nursing.** By ISABEL ADAMS HAMPTON.

"It is not too much to say that in no single volume yet published has the subject been so scientifically, carefully, and completely treated as it has been by Miss Hampton. . . . Whether as a teaching manual or as a book of reference, the thoroughly practical instruction which it conveys is sure to obtain a wide circulation."—*The Hospital.*

Demy 8vo, cloth extra, price 3s. 6d. (post free). Popular Edition, stitched, price 1s. 6d.

**Outlines of Insanity.** A Popular Treatise on the Salient Features of Insanity. By FRANCIS H. WALMSLEY, M.D., Medical Superintendent of the Darenth Asylum, Metropolitan Asylums Board, Member of Council of Medico-Psychological Association.

A clearly written manual in which the various features of mental disorder are fully expounded. The author's style is interesting and the reader's attention is riveted throughout.

Crown 8vo, cloth gilt, price 2s. 6d.

**The Mother's Help and Guide to the Domestic Management of Children.** By P. MURRAY BRAIDWOOD, M.D., Formerly Senior Medical Officer to the Wirral Hospital for Sick Children.

Just out, crown 8vo, cloth gilt, Illustrated, price 5s.

**Helps in Sickness and to Health:** Where to Go and What to Do. Being a Guide to Home Nursing and a Handbook to Health in the Habitation, the Nursery, the Schoolroom, and the person, with a chapter on Pleasure and Health Resorts. By HENRY C. BURDETT, Author of "Hospitals and Asylums of the World," "Pay Hospitals of the World," "Cottage Hospitals, General, Fever, and Convalescent," &c., &c.

"It would be difficult to find one which should be more welcome in a household than this unpretending but most useful book."—*The Times.*

LONDON: THE SCIENTIFIC PRESS, LTD., 428, STRAND, W.C.

